# Adolescents’ lived experiences of substance abuse in the Greater Giyani Municipality

**DOI:** 10.4102/curationis.v46i1.2379

**Published:** 2023-01-31

**Authors:** Annie Temane, Tinyiko Rikhotso, Marie Poggenpoel, Chris Myburgh

**Affiliations:** 1 Department of Nursing, Faculty of Health Sciences, University of Johannesburg, Johannesburg, South Africa; 2 Department of Educational Psychology, Faculty of Education, University of Johannesburg, Johannesburg, South Africa

**Keywords:** adolescents, lived experiences, substance abuse, mental health, mental healthcare professionals

## Abstract

**Background:**

Adolescence is a unique and distinct stage of development that involves changes in the physical, psychological and social aspects of adolescents. It is a critical transition into adulthood whereby heightened risk-taking and sensation-seeking takes place, such as substance abuse. In a South African context, this transition sometimes occurs under economic stress, poverty, unemployment, high levels of crime and political instability. This can place adolescents at risk of substance abuse.

**Objectives:**

To explore and describe the lived experiences of adolescents abusing substances in the Greater Giyani Municipality in the Limpopo province, South Africa.

**Method:**

A qualitative, exploratory, descriptive and contextual research design with a phenomenological approach was used. Data were collected through individual, in-depth, phenomenological interviews and field notes. Thematic coding was utilised to analyse the collected data, and literature was reviewed to support the findings. Moreover, measures to ensure trustworthiness and ethical principles were applied throughout the research process.

**Results:**

Five themes were identified: substance abuse behaviour among adolescents, adolescents’ motivation for continuing substance abuse, the effects of substance abuse on the lives of adolescents, factors affecting adolescents’ discontinuation of substance abuse and a need to discontinue substance abuse.

**Conclusion:**

The study concluded that adolescents abusing substances in the Greater Giyani, Limpopo province, experience loss of control, broken relationships, poor academic performance, stigma attached to mental illness and negative emotions. The adolescents foresaw their future as uncertain and without direction. It is recommended that mental healthcare professionals introduce and implement interventions that will assist the adolescents who abuse substances in the Greater Giyani, Limpopo province.

**Contribution:**

The findings in this study could add knowledge in developing and implementing of strategies for psychiatric nurses to support adolescents abusing substances in the Greater Giyani, Limpopo province.

## Introduction

According to the World Health Organization (WHO 2014:15), there is an increased percentage of adolescents abusing substances in communities at large, regardless of the support that parents, caregivers and healthcare professionals are providing to reduce the social problem. Despite the fact that substance abuse is a problem experienced by all age groups, adolescents are particularly among the most affected (Hlungwani et al. 2020:1).

Moen and Hall-Lord (2019:1) explain that the adolescent period is the transition process to adulthood and is characterised by significant mental and physical changes. This is in addition to individual, social and contextual transitions that occur in the adolescent period. This transition sometimes occurs within a context of economic stress, poverty, unemployment, high levels of crime and political instability, placing adolescents at risk of using substances (Smart 2019:118). The WHO (2022:1) defines an adolescent as a person between the ages of 10 and 19 years. Salmela-Aro (2011:360) describes an adolescent according to stages: early adolescence (11 to 13 years); middle adolescence (14 to 18 years); and late adolescence (19 to 21 years). Thus, in this study ‘adolescents’ will refer to young persons between the ages of 18 and 21 years.

Radebe (2015:6) states that poverty and economic challenges, such as in developing countries such as South Africa, can indirectly contribute to the psychosocial implications of substance abuse. In addition, the consequences of substance abuse have an impact on family members and communities (Hemovich, Lac & Crano 2011:10). Radebe (2015:6) further indicated that tolerant parental attitudes and sibling substance use are the family factors most strongly associated with an increased prevalence of binge drinking, cigarette smoking, cannabis use and illicit drug use among adolescents.

The use and abuse of substances, especially among the youth, continues to be a serious concern within the international community (Alhyas et al. 2015:1). The use and abuse of substances affects the adolescents, their families, the community and society at large. This is evident when such practices cause social and family disorganisation, economic instability and social insecurity and curb progress for individuals, families and the state. Some effort towards intervening in this challenge has existed and continues to exist, yet it appears insufficient (Matheson et al. 2022:3). There are some shortfalls in interventions, as no satisfying improvements have been reported; instead, the prevalence of substance abuse is becoming worse each day (Cardenas et al. 2021:91).

Interventions, as reported by Salam et al. (2016:6), considered for prevention of substance abuse include, but are not limited to, promotion of sexual and reproductive health, nutritional interventions and mental health promotion. Malik (2020:590–594) conducted a case study in the United States on a 17-year-old boy who was abusing substances, which led to altered behaviour and unhealthy relationships. Their interventions focused on enhanced communication skills, emphasising the consequences of untoward behaviour, minimising associations with substances, using a stimulating environment, training to improve resistance skills and controlling procedures to reduce the desire to engage in substance use. Progressively, there was a marked improvement in behaviour and relating to others, as well as a reduction in the adolescent’s level of substance use.

As the researcher, a professional nurse at the time of this study, engaged in routine activities in a public psychiatric hospital in the Greater Giyani Municipality in Limpopo province, South Africa, she realised that most adolescents admitted to the psychiatric ward had a history of abusing substances. Some adolescents were diagnosed as having substance use disorder (SUD), and others’ substance use was the underlying trigger to mental conditions, such as bipolar mood disorders and schizophrenia. The National Institute of Mental Health (NIMH) (2022:np) defines SUD as a mental disorder that affects a person’s brain and behaviour, leading to inability to control the use of substances such as legal or illegal drugs, alcohol or medications. This leads to addiction and consequent recurrent relapses. Studies on the subject of adolescent substance abuse (Lebese, Ramakuela & Maputle 2014:336; Malik 2020:590–594; National Drug Master Plan [NDMP] 2014–2019:52; Sadock, Sadock & Ruiz 2017:1100; Salam et al. 2016:6; WHO 2014) have been conducted, but none explored the adolescents’ lived experience of substance abuse in the Greater Giyani Municipality, Limpopo province, South Africa.

The primary beneficiaries of this study are adolescents abusing substances and psychiatric nurses caring for these adolescents. The findings drawn from this study can contribute to the body of knowledge for the nursing profession regarding the lived experiences of adolescents abusing substances. This will ultimately increase nurses’ understanding (especially psychiatric nurses) of the behaviour adolescents display and enable them to offer necessary care and support. It will also inform policymakers on aspects to include during policy formulation on adolescent substance abuse. It is anticipated that the findings will evoke further research studies around the same subject. There is a need to describe the lived experiences of adolescents abusing substances in the Greater Giyani Municipality so psychiatric nurses can provide support and promote their mental health.

### Aim

The study’s objective was to explore and describe adolescents’ lived experiences of substance abuse in the Greater Giyani Municipality of the Limpopo province.

## Methods

### Study design and methods

A qualitative, exploratory, descriptive and contextual (Gray & Grove 2020:359) research design, with a phenomenological approach, was utilised in this study. The descriptive phenomenological approach (Creswell & Creswell 2017:79) allowed for capturing the lived experiences of adolescents abusing substances.

### Setting

The research setting was a public psychiatric hospital in the Greater Giyani Municipality in the Limpopo province, which is situated in the northern part of South Africa. Greater Giyani is a rural area and falls under the Greater Giyani Municipality; it is one of five local municipalities under the Mopani District Municipality. Participants were from various villages around Greater Giyani. The selected public psychiatric hospital has 10 wards with 354 beds; 235 beds were in use at the time of data collection, with an average of 130 adolescents admitted at the hospital. The hospital admits mental healthcare users with different mental health disorders and provides assisted and involuntary inpatient care, treatment and rehabilitation services.

### Study population and sampling strategy

The study’s population was adolescents admitted to this public psychiatric institution. Purposive sampling (Creswell & Creswell 2017:121) was utilised to select adolescents abusing substances. The inclusion criteria were adolescents between 18 and 21 years of age, admitted to a public psychiatric hospital in Greater Giyani Municipality, who could speak either in English or Xitsonga as the commonly used languages in the province and were willing to provide information about their personal experiences of abusing substances such as marijuana, inhalants, ‘*nyaope*’ and alcohol, which are commonly used substances in the Greater Giyani area. *Nyaope* is an illegal cocktail drug particular to South Africa; it is a mixture of antiretroviral drugs, rat poison, acid and detergents to produce a powder (Bala & Kang’ethe 2021:307).

According to Patino and Ferreira (2018:84), exclusion criteria are characteristics of potential study participants who meet the inclusion requirements but have extra traits that could hinder the study’s success or raise the likelihood of an adverse result. The exclusion criteria of this study were adolescents who were not admitted at a public psychiatric hospital and those who were admitted, but their condition was acute, and they presented with psychotic features.

### Data collection

Data were collected at a public psychiatric hospital in the Greater Giyani Municipality in the Limpopo province. In-depth, individual, phenomenological interviews and field notes (Mavhandu-Mudzusi 2018:1–9) were used to collect data on adolescents who had lived experiences of abusing substances in the Greater Giyani Municipality of Limpopo province. The interviews were conducted from August 2007 to October 2007. Although both boys and girls met the inclusion criteria, only boys were available at the time of study, as boys are prone to substance abuse in Greater Giyani Municipality, Limpopo province. One central question was asked: ‘How was it for you to use drugs?’ The participants’ permission was requested to audio-record the interviews, which lasted 40 min – 60 min. Data were collected until data saturation (Gray & Grove 2020:361) was reached at the eighth interview, as evidenced by repeating themes.

### Data analysis

Audio-recordings were transcribed verbatim, and transcriptions and field notes were analysed through thematic coding, according to the steps suggested by Creswell and Creswell (2017:137). An independent coder (an expert in qualitative data analysis) assisted in co-coding with the researcher. She received a clear set of transcripts and field notes for analysis. The researcher and the independent coder had a consensus discussion about relevant themes and categories of adolescents’ lived experiences of abusing substances. The findings were ultimately contextualised within the literature.

### Trustworthiness

The researcher implemented Guba’s model of trustworthiness (Denzin & Lincoln 2018:1380) and focused on aspects of credibility, transferability, dependability and confirmability to ensure the quality of the study. To promote credibility, the researcher ensured prolonged engagement by engaging with participants for about 3 months. Questions were correctly phrased, and clarifications were sought where necessary. Triangulation of data sources, through the use of audio-recordings, in-depth phenomenological interviews, observations and field notes, was promoted (Fouche, Strydom & Roestenburg 2021:419). In order to ensure the study’s transferability to similar contexts, the researcher used purposive sampling to obtain relevant information from participants. Demographic data, such as age, gender, cultural and religious practices and participants’ level of education, were described. Participants’ responses to questions were analysed and described, supported by direct quotes and relevant literature. The researcher also densely described the research methodology (Polit & Beck 2018:511) to ensure the study’s dependability. The research process included the population and sampling, which was purposive. Data were collected through phenomenological interviews and field notes. Moreover, data were analysed using thematic coding, and a description of the findings was provided. Each step was supported by literature, with appropriate references. To ensure confirmability, the researcher, supervisors and independent coder analysed the data and discussed the findings until they reached a consensus.

### Ethical considerations

Approval and permission to conduct the study were obtained from the Faculty of Health Sciences Academic Ethics Committee of the University of Johannesburg (ref. no. REC-01-169-2016); the National Health Research Database in Limpopo province; the Department of Health, Limpopo province; and the institution in which the study was conducted.

Ethical principles that were considered for the study were autonomy, nonmaleficence, beneficence and justice (Dhai & McQuoid-Mason 2011:134). In this study, all these principles were adhered to throughout the research process. The principle of autonomy was respected by treating participants as autonomous agents with the freedom to volunteer their consent to participate in the study. The right to anonymity and confidentiality was maintained by not utilising any identifiers of the adolescents in the study, and they were also assured of the latter. The collected data were documented and kept safe (Corbin & Straus 2015:345). The raw data (in the form of audio-recordings, transcriptions and field notes) were kept safe in a locked cupboard and only accessed by the researcher, the supervisor and the cosupervisor (Polit & Beck 2018:336).

Adolescents abusing substances were informed about the study’s aims, the option to participate and the right to withdraw at any time without being penalised (Polit & Beck 2018:124). Written consent and permission to audio-record the interviews was obtained from the adolescents to participate in the study. To ensure the principle of beneficence, the researcher indicated to the adolescents that there were no direct benefits in participating in the research. However, to uphold the principle of nonmaleficence, the researcher was sensitive to any signs of emotional discomfort from participants to avoid harm and risk to their emotional well-being.

In order to ensure the principle of justice, the researcher guaranteed the participant’s right to fair selection and the right to fair treatment. The sampling criteria were utilised as a measure to implement the principle of justice. Polit and Beck (2018:124) state that the selection of research participants should be based on research requirements and not on their vulnerability status.

## Findings

### Demographic profile of the participants

The participants were male adolescents and were admitted in a public psychiatric hospital in the Greater Giyani. Their ages ranged from 18 to 21 years. Seven of the participants were fluent and comfortable communicating in English, and only one requested to express himself in his own African language, Xitsonga. At the time of the interviews, three participants were in Grade 10, two in Grade 11 and two in Grade 12, and one was in the first year of tertiary education at a college. The participants have been using substances for 3–6 years. All of the participants were living with their parents. Table 1 presents the demographics of the participants.

**TABLE 1:** Participants’ demographics.

Participant	Gender	Age (years)	Substance abused	Number of years using the substance
Participant 1	Male	18	Alcohol	3
Participant 2	Male	20	Alcohol, marijuana	6
Participant 3	Male	19	Marijuana, *nyaope*	5
Participant 4	Male	18	Alcohol, glue	5
Participant 5	Male	19	Alcohol, marijuana, *nyaope*	4
Participant 6	Male	19	Alcohol, marijuana	5
Participant 7	Male	21	Glue	5

*Source*: Rikhotso, T.N., 2019, ‘Substance abuse among adolescents in the Limpopo Province’, Mcur dissertaion, University of Johannesburg

### Presentation of findings

This section presents participants’ experiences under the following five themes: (1) adolescents’ substance abuse behaviour; (2) adolescents’ motivation for continuing substance abuse; (3) the effects of substance abuse on the lives of adolescents; (4) factors affecting adolescents’ discontinuation of substance abuse and (5) a need to discontinue substance abuse.

The themes are summarised in Table 2. Thereafter, the findings are discussed.

**TABLE 2:** Themes of adolescents’ lived experiences of substance abuse.

Themes
1. Adolescents’ substance abuse behaviour
2. Adolescents’ motivation for continuing with substance abuse
3. Effects of substance abuse on the adolescents’ life
4. Factors affecting adolescents’ discontinuation of substance abuse
5. A need to discontinue substance abuse

*Source*: Rikhotso, T.N., 2019, ‘Substance abuse among adolescents in the Limpopo Province’, Mcur dissertaion, University of Johannesburg

#### Theme 1: Adolescents’ substance abuse behaviour

In all the transcribed interviews, adolescents who abused substances reported they were groomed to pursue activities that eventually led to a habit of substance abuse. In concurrence with the findings, the participants expressed the following:

‘I decided to drink beer and get drunk so that I will not be afraid to ask my mother who my real father was.’ (Participant 5)

Adolescents commented:

‘I decided to make friends with those boys and started smoking and drinking a little bit. I gradually got used to it because I did not want to lose my friends.’ (Participant 2)

‘I tried to imitate what daddy was doing when he was smoking.’ (Participant 8)

‘Now when I am not in hospital like I am at present, I drink almost every day.’ (Participant 4)

Adolescents were also initiated into substance abuse through peer groups and curiosity about the effects of certain substances. Adolescents were eager to explore and learn about new things; they wanted to fit in and not feel isolated by their peers. This is seen in the statements presented below:

‘I put the “stompies” together until I manage to make a big tobacco wrap, which together with boys of my age from the neighbourhoods got to a corner and smoked.’ (Participant 3)

‘My friends who were already smoking and drinking started laughing at me because I was the only person in the group who was not smoking.’ (Participant 5)

‘He said extending his arm that I should have a taste of it [*glue*] by sniffing and see what will happen.’ (Participant 7)

From the collected data, it is evident that the adolescents were determined to experiment and explore the substances they were exposed to. Participants explained:

‘I wanted to experiment with them myself and I eventually got used to them.’ (Participant 3)

‘Later when he left, I told myself I want to taste it again.’ (Participant 4)

Parents’ modelling and values also seemed to contribute to the adolescents’ substance abuse. One of the adolescents said:

‘I began when my father started buying us liquor during special holidays such as Christmas, Easter, New Year and during ancestral celebrations at home. He used to tell us that he is buying us the beer and want us to enjoy just for the occasion.’ (Participant 8)

Another said:

‘My father is a heavy smoker and he used to smoke in front of me. All along when he was smoking I was wondering how he was doing it when I saw some smoke coming out through his mouth and nostrils. He appeared to be enjoying when he was smoking and I admired him.’ (Participant 5)

Most of the adolescents who participated in the study found the means to acquire the substance they ultimately abused. Participants shared:

‘I accumulated that pocket money until it was more enough to buy beer whenever I felt like drinking.’ (Participant 6)

‘When it comes to a push, I even steal some coins at home to make sure I go to buy some glue.’ (Participant 8)

The above statements reflect a number of factors contribute to the initiation and continued use of substances. These include parental influences, self-medication and peer pressure, among others (Broman 2016:491).

#### Theme 2: Adolescents’ motivation for continuing with substance abuse

Adolescents were motivated by some internal and external influences to continue abusing substances. They also used substances as a means of addressing emotional and social challenges. Moreover, during the interviews, adolescents appeared afraid of being rejected by their peer group members. The following statements confirm this finding:

‘I need to tell my friends that I don’t want to drink again and say no when they invite me to take rounds with them, but I still feel that they will laugh at me and I might lose them.’ (Participant 1)

‘If I quit smoking my friends will laugh at me saying I am a coward. If I make friendship with non-smokers they will reject me and say I want to teach them my bad behaviour. So, it means I am in the middle. I don’t know where I belong anymore.’ (Participant 8)

It is evident from the above statements that the adolescents had a desire to quit substance abuse but were not ready to lose the relationships they had with their friends. Moreover, the adolescents who abuse substances are also often fearful of being victimised when they withdraw from the group with which they used to smoke or drink.

One participant explained:

‘This time they were a bit harsh and I could see that my life was in danger.’ (Participant 4)

‘If I decide to leave them even if I could, these guys will kill me thinking that I will reveal their secret to the police.’ (Participant 2)

‘*Embilwini ya mina a ndzi swi navela ku va byela leswaku ku dzaha mbangi swi bihile kambe a ndzinga ta swi kota hikuva papa a va lava les waku hi vuyisa mali leyi hi nga xavisa mbangi masiku hikwawo*.’ [In my heart I did desire to tell that smoking dagga is a bad habit, but I couldn’t because my father wanted us to bring money home every day.] (Participant 3)

These statements illustrate that adolescent substance abusers wanted to do well, but they could not for fear of being placed in danger, so they continued with the habit.

Challenges, such as feelings of loneliness, decreased energy and the need to numb emotional pain, are experienced, as expressed in the following statements:

‘I sometimes could crave for liquor when I am lonely and during weekends.’ (Participant 8)

‘I drank beer and get drunk and then I would not be afraid of asking my mother who my real father is.’ (Participant 1)

‘In my spare time I used to drink beer telling myself that maybe I will forget.’ (Participant 3)

‘All what I have explained made me frustrated and I started drinking, thinking that it will help me forget all that was happening.’ (Participant 6)

‘What I have discovered is that even if I get drunk, when I’m sober the problems and frustrations remain.’ (Participant 4)

It is indicated in the latter statements that adolescents attempted to deal with depression, pain (physical or emotional) or intense emotions with the help of alcohol and other substances.

#### Theme 3: Effects of substance abuse on adolescents

The adolescents felt powerless and confused. These are described as follows:

‘I don’t know what to do to solve this problem.’ (Participant 5)

‘I am confused and don’t know what I can do to come out of this mess.’ (Participant 8)

‘This whole thing makes me left in the middle without knowing what to do to come out of this problem.’ (Participant 3)

The adolescents’ willpower to decide what to do in a challenging situation had been markedly affected. The above statements confirm that the adolescents found it difficult to make decisions regarding the challenging situations they encountered. Participants were also affected psychologically and expressed the following reactions:

‘I also feel hopeless as I tried many times to quit drinking, but I am failing.’ (Participant 3)

‘I sometimes feel it was better if I was dead because my whole life is destroyed.’ (Participant 4)

‘I am overwhelmed by feelings of guilt, shame and feel hopeless.’ (Participant 7)

It is evident from the presented statements that the adolescents were emotionally affected and expressed feelings of loss, isolation and being rejected in terms of interpersonal relationships. They explained:

‘My father does not trust me anymore.’ (Participant 2)

‘My girlfriend is also threatening to leave me.’ (Participant 5)

Adolescents’ abuse of substances affects their lives as individuals, their families and the community at large. People in these adolescents’ vicinity are similarly affected in different and unique ways (Lander, Howsare & Byrne 2013:194–205).

‘My father is deserting me and is blaming my mother for not doing enough to prevent me from doing the drugs.’ (Participant 6)

‘I am not happy if my own sister is afraid of me.’ (Participant 7)

The relationships between adolescents abusing substances and their families and friends were negatively affected, as the latter statements concur.

#### Theme 4: Factors affecting adolescents’ discontinuation of substance abuse

Several factors that negatively or positively influence adolescents’ ability to discontinue the abuse of substances were identified. These included aspects of addiction, motivation to discontinue and insight into substance abuse. During the interviews, it was noted that the adolescents abusing substances had lost control over their own lives; instead, the substances they abused controlled them.

‘I see myself as a slave of beer and I want it with all my heart to quit drinking.’ (Participant 1)

‘But since I started sniffing glue, I cannot control it. I frequently crave for it and make sure I get it.’ (Participant 4)

‘I am now a slave of drugs and can’t do without them.’ (Participant 7)

Adolescents’ experiences with substance abuse ultimately triggered their motivation to cease the behaviour. The following statements support this finding:

‘I have many things to do than to come and stay here in hospital. I don’t like being in this place. Some people who are here are madder than me, so I don’t feel good staying with them. I will try by all means to avoid being brought here again by doing away with these drug things.’ (Participant 3)

‘I can be very happy if I can be able to leave sniffing glue. I don’t want to come to this place again. It was better if I came because my body was sick, not my mind.’ (Participant 8)

These latter statements confirm the participants did not want to continue their substance abuse behaviour based on their lived experiences. They had the desire to quit.

‘I am addicted to drinking and I am told it is one of the reasons I am admitted in hospital because it has affected my mind. It may be true because now I drink more frequently.’ (Participant 3)

‘It is wrong because I am still underage. I just did it in order to get the truth from my mother and now I realised I used the wrong way.’ (Participant 5)

‘It is a bad habit. It is just that once a person starts using drugs, it is not simple to leave them.’ (Participant 7)

Evidently, the adolescents gained an understanding of the negative effects imposed on them by the abuse of substances and gained insight into the effects of substance abuse on their lives. Their understanding of these effects heightened their level of judgement and decision-making ability.

#### Theme 5: A need to discontinue substance abuse

Most adolescents expressed the need to discontinue substance abuse by cutting off relationships and situations that triggered this behaviour. The adolescents in this study revealed an understanding that their continued avoidance of substances means certain people and places have to be avoided. This finding is reflected in the direct quotes below:

‘I have something in mind. I am just not sure if it is going to work well for me. I was thinking of breaking the friendship with people I used to smoke with. Even if they invite me to go with them, I will not agree. I will tell them straight that I don’t belong to their group anymore.’ (Participant 4)

‘I plan that when my friends invite me to take rounds, I will tell them I am busy with something else. I will try to reduce the number of cigarettes I used to smoke in a day bit by bit. I heard that there are nicotine tablets that can help quench the craving; I will ask my father to get them for me from the chemist.’ (Participant 6)

The adolescents were aware of the benefits linked with quitting substance abuse, especially when they made comparisons with the challenges they encountered as a result of abusing these substances. This led them to a turning point where they realised the need to stop their destructive behaviour (Pettersen et al. 2018:np). All the adolescents abusing substances who were interviewed mentioned the need to terminate their use based on the negative influence these substances had on their lives. Although they still doubted the success of their plan to quit, they were determined to give it a try. Participants shared:

‘I am afraid of those guys. I was planning to leave the group, but I fear that if I do this while studying in the same institution and residing in the same place which they know, I may lose my life.’ (Participant 3)

‘*Ndzi na makungu yo karhi. Ku fika sweswi ndza chava ku endla makungu lawa. Ndzi chava papa mina. Rin’wana ra makungu ya kona I ku pota papa emaphoriseni leswaku va xavisa mbangi na leswaku va xanisa vandyangu lavan’wana*.’ [I do have some plans. So far, I am still afraid to pursue those plans. I am afraid of my dad. One of those plans was to report him to the police for being a dagga dealer and for abusing my other family members.] (Participant 5)

The adolescents in this study also expressed the same feelings of fear related to the discontinuation act itself.

## Discussion of findings

Multiple challenges were experienced by participants in this study; the challenges include being influenced to abuse substances by the environment they live in, having the motivation to quit but being fearful of losing relationships with peers or, for some, with parents. Adolescents were affected by substance abuse as they wanted a bright future for themselves but were having difficulty quitting. The study findings are discussed below.

In this study, participants shared they were influenced by several factors, which included peer pressure and family modelling the behaviour, such as alcohol use in the home. This is supported by findings from a study by De Witt (2016:316), stating that adolescents want to explore and know more about things around them. In addition, Broman (2016:491) states that parental influence, self-medication and peer pressure, among others, are factors that contribute to the initiation and continued use of substances.

Adolescents in this study reported that they were motivated to continue using substances because of challenges they were facing. Participants reported being rejected by their peers, and they wanted to fit in. To counteract those feelings, individuals might be compelled to continue with undesired behaviours to gain the support of those to whom they are attached (Leary 2015:435–441). Nebhinani et al. (2012:155) also agree with these findings, stating that the adolescents had a desire to cease their substance abuse behaviour but were simultaneously afraid of losing peer relationships, so they could not stop. Moreover, adolescents in this study reported feelings of loneliness and frustration and wanted to forget about the challenges they were facing. Esan et al. (2018:1–7) concur with this finding, stating self-medication was also increasing adolescents’ risk of drug abuse and drug dependence. In this instance, self-medication relates to participants using substances to numb the emotional and psychological pain they are experiencing.

Findings from the study indicated that adolescents found it difficult to make decisions that impacted their lives; this is supported by Frith (2013:289–299), who is of the opinion that volition is an intentionally and internally binding action that is voluntarily taken to ensure the desired outcome is fulfilled.

Emotions are complex psychological processes involving multiple response channels, including physiological systems, facial and vocal expressive tendencies and cognition. These channels influence each other in a process that extends over time (Kassam & Mendes 2013:1). Adolescents in this study experienced the loss of interpersonal relationships and family members, and the community had lost trust and faith in the adolescents; this negatively affected adolescents. People in these adolescents’ vicinity were similarly affected in different and unique ways (Lander et al. 2013:194–205). The relationships between adolescents abusing substances and their families and friends were negatively affected.

Participants in this current study had lost control of their lives; they found it difficult to refrain from the substances. It has been determined that adolescents often have difficulty refraining from addictive behaviour despite attempting to do so because of the ‘loss of control’ aspect of addiction (Sussman & Sussman 2011:4025–4038).

According to Pettersen et al. (2019:6), a change of the environment the adolescent is subjected to is important for initiating and maintaining abstinence from substance use. Similarly, the adolescents in this study revealed an understanding that their continued avoidance of substances means certain people and places have to be avoided.

The adolescents were aware of the benefits linked with quitting substance abuse, especially when they made comparisons with the challenges they encountered as a result of abusing these substances. Pettersen et al. (2018:np) state that when adolescents are aware of the benefits of abstinence, it leads them to a turning point where they realise the need to stop their destructive behaviour. All the adolescents abusing substances who were interviewed in this study mentioned the need to terminate their use based on the negative influence these substances had on their lives. Although they still doubted the success of their plan to quit, they were determined to give it a try.

In conclusion, the adolescents’ fear of disappointing others, losing social support, peers’ reactions and treatment failures were the main sources of fear surrounding adolescents’ discontinuation of substance abuse (Nebhinani et al. 2012:155–158). The adolescents in this study also expressed the same feelings of fear related to the discontinuation act itself.

## Limitations

Some of the adolescents admitted to the psychiatric unit with a history of abusing substances were still quite psychotic when the interviews were conducted. Their lived experience would have added value to the study should they have been stable enough to share. Other participants preferred to express themselves in English, yet they could not find the relevant vocabulary to express their situations accurately. In the process, some important information was missed. Also, since the research context was a short-term psychiatric unit, adolescents would sometimes be discharged before follow-up sessions could be conducted; hence, some information was missed. All the participants were boys, and having girls interviewed on their substance abuse experiences could have been beneficial as input from both genders would have been obtained.

## Recommendations

### Nursing practice

Psychiatric nurse practitioners need to be sensitive when dealing with adolescents with a history of substance abuse. The adolescents must be treated with caution and respect so that they feel accepted. Thus, it is important for psychiatric nurse practitioners to utilise effective communication skills to explore the complexity of the problem and create an environment that encourages adolescents to uncover their real problems. In addition, psychiatric nurse practitioners need to support and assist adolescents in making their own decisions and developing their own insight (Jones, Fitzpatrick & Rogers 2016:70).

### Nursing education and mental health education

The study’s findings indicated that mental health could be promoted by providing mental health education to assist adolescents in dealing with day-to-day life challenges. Nurse educators can promote mental health education in the curriculum to teach learners on substance abuse disorders related to adolescents. Adolescents need support from psychiatric nurses and their parents in order to achieve optimal mental functioning; therefore, the nursing education curriculum should focus on improving mental health education in communities and communication skills. Emphasis needs to be placed on community-based care to highlight adolescents’ needs related to the resources available in the community.

### Nursing research

Further studies should be conducted to ensure psychiatric nurses facilitate the mental health of adolescents abusing substances. There is a need for additional studies in the Greater Giyani Municipality in the Limpopo province, exploring the lived experiences of adolescents abusing substances.

## Conclusion

The findings indicate that adolescents who abuse substances in the Greater Giyani experience challenges that can have an impact on their body, mind and spirit. In addition, the findings reveal that adolescents using substances need support from family, the community, as well as mental healthcare professionals. It is thus recommended that mental healthcare professionals introduce and implement relevant interventions such as support groups, mental health education, mental health awareness campaigns and rehabilitation services that directly deal with substance abuse, and the implementation thereof needs to be monitored and evaluated.
